# Creative metacognition in design thinking: exploring theories, educational practices, and their implications for measurement

**DOI:** 10.3389/fpsyg.2023.1157001

**Published:** 2023-05-09

**Authors:** Julia P. A. von Thienen, Theresa J. Weinstein, Christoph Meinel

**Affiliations:** Hasso Plattner Institute, Digital Engineering Faculty, University of Potsdam, Potsdam, Germany

**Keywords:** accuracy, creativity, design thinking, education, measurement, metacognition, innovation, framework

## Abstract

Design thinking is a well-established practical and educational approach to fostering high-level creativity and innovation, which has been refined since the 1950s with the participation of experts like Joy Paul Guilford and Abraham Maslow. Through real-world projects, trainees learn to optimize their creative outcomes by developing and practicing creative cognition and metacognition. This paper provides a holistic perspective on creativity, enabling the formulation of a comprehensive theoretical framework of creative metacognition. It focuses on the design thinking approach to creativity and explores the role of metacognition in four areas of creativity expertise: Products, Processes, People, and Places. The analysis includes task-outcome relationships (product metacognition), the monitoring of strategy effectiveness (process metacognition), an understanding of individual or group strengths and weaknesses (people metacognition), and an examination of the mutual impact between environments and creativity (place metacognition). It also reviews measures taken in design thinking education, including a distribution of cognition and metacognition, to support students in their development of creative mastery. On these grounds, we propose extended methods for measuring creative metacognition with the goal of enhancing comprehensive assessments of the phenomenon. Proposed methodological advancements include accuracy sub-scales, experimental tasks where examinees explore problem and solution spaces, combinations of naturalistic observations with capability testing, as well as physiological assessments as indirect measures of creative metacognition.

## 1. Introduction

Metacognition, often referred to as “thinking about thinking,” encompasses the monitoring and control of one’s own thought processes (Flavell, 1987; Papaleontiou-Louca, 2003; Martinez, 2006). It is second-order cognition, with individuals engaging in reflections about their own thoughts and knowledge. In practical contexts, metacognition often manifests as reflections and beliefs about one’s past, present, or potential future courses of action.

Flavell (1987), a pioneer in metacognition studies, emphasizes that having metacognitive abilities is crucial for organisms with the following traits: They (i) think a lot (ii) in error-prone ways; they face tasks of (iii) explaining and justifying their thinking to others and themselves, they (iv) have to plan ahead and critically evaluate alternative options, (iv) making weighty decisions in carefully pondered ways. He notes: “Needless to say, *human beings* are organisms with just these properties” (p. 27, emphasis in original).

Creativity is a domain where the above characteristics are particularly relevant, and therefore metacognition plays an especially prominent role. Creativity aims for products, or problem-solutions, that are *novel* and *valuable* (Runco and Jaeger, 2012; Paul and Kaufman, 2014; Royalty and Roth, 2016). The degree to which products are novel and valuable defines the degree of creative achievement (Boden, 2004; Kaufman and Beghetto, 2009; Royalty and Roth, 2016). Minor creative achievements, referred to as “mini-c,” are new and valuable only to the creator. By contrast, eminent creative achievements, referred to as “Big-C” or “H-creativity,” are new and valuable to humanity and have not been previously seen in human history. Additionally, there are levels of creative achievement between mini-c and Big-C, such as “little-c,” in which the solutions are new and valuable to a specific audience like peers or one’s family, and “pro-c,” in which solutions are appreciated as new and valuable within an expert community.

It is important to note that exceptional levels of creative accomplishment involve pushing the boundaries of knowledge beyond what is currently known to humanity (Boden, 2004; von Thienen et al., 2017). This requires venturing into uncharted territory, which is more error-prone compared to re-using established knowledge and taking small steps in well-explored fields. High-level creative performance is challenging. Therefore, most people are only creative at lower levels of accomplishment, and only a few people become eminent creators. Furthermore, when creators work on subjects that matter to humanity, such as designing a policy to promote peace and societal flourishing, they are faced with weighty decisions. This stands in contrast to situations of more mundane creativity at lower levels of accomplishment, such as scribbling arbitrary drawings on paper, which hardly anyone cares about, and where no particularly weighty decisions need to be made. Thus, especially at high performance levels, creative activity introduces the exact conditions listed by Flavell (1987), where organisms benefit from metacognition.

Against such backgrounds, the concept of metacognition has garnered increasing attention in the realm of creativity studies, leading to the coinage of the term “creative metacognition.” Kaufman and Beghetto (2013) define it as “a combination of creative self-knowledge (knowing one’s own creative strengths and limitations, both within a domain and as a general trait) and contextual knowledge (knowing when, where, how, and why to be creative)” (p. 160). This definition regularly serves as a reference point in research (e.g., Kaufman et al., 2016; Puente-Díaz and Cavazos-Arroyo, 2022; Puente-Díaz et al., 2021). While this definition is conductive for studying the role of metacognitive knowledge about one’s creative self and context, we argue that an even more comprehensive and systematic theoretical framework is needed to capture the phenomenon of creative metacognition in all its facets. In this paper, we introduce a systematic framework of creative metacognition that covers four competency domains. It is concerned with metacognition on creative ***P***roducts, ***P***rocesses, ***P***eople, and ***P***laces. Overall, we will build on the following understanding: *Creative metacognition is thinking about creative thinking with the aim of improving creative performance, or enhancing creative capacity*. This definition highlights the role that metacognition plays in creative practice and education: Trainees learn to improve their creative metacognition in order to become better creators. Similarly, professional creators engage in metacognition in order to correct errors, take good decisions, and to develop a deeper understanding of creativity as an improved foundation for future work.

In recent discourse, it has been noted how metacognition is specifically important, but also specifically challenging to study in the context of creativity. As Puente-Díaz and Cavazos-Arroyo (2022) point out, “we need to go beyond paying attention to creative cognition and also examine creative metacognition. This is particularly relevant given that creativity problems are ill-defined and open-ended” (p. I). The measurement of creative metacognition is highly relevant for creativity research and education alike, for instance as a means to assess cognitive differences between novice and expert creators (cf. Dorst and Reymen, 2004). However, as Puente-Díaz and Cavazos-Arroyo (2022) note, assessing metacognition in the context of creativity presents methodological difficulties. In the case of creative work, people tackle open-ended problems that have many potential solutions ranging from better to worse. These problems contrast to cases where a single correct answer exists, with math tasks providing classic examples, or challenges where performance is optimized on a specific dimension, such as providing the greatest number of correct answers in a multiple-choice knowledge test under time constraints. Sidi et al. (2020) have summarized metacognition studies that were conducted over time, observing that for the most part research used to focus on well-defined problems having right-or-wrong answers. By comparison, metacognition research concerning ill-defined problems is a relatively young scientific field facing methodological challenges as it is just taking shape.

This paper examines creative metacognition as practiced in the context of design thinking, which is a practical approach for the training of professional creators, aiming at the high-end spectrum of creative performance. With this paper, we seek to contribute toward systematic grounds for the development of assessment approaches that quantify different aspects of creative metacognition. One key objective is to more accurately represent the entirety of the phenomenon. For this purpose, we review the theory, educational practice and research of design thinking. The training of metacognition plays a central role in this approach, and sophisticated training interventions exist for this purpose. The paper introduces design thinking and then discusses metacognition as trained in four domains (“4P”), covering creative ***P***roducts, ***P***rocesses, ***P***eople, and ***P***laces. It outlines different approaches in design thinking education, including a distribution of cognition and metacognition to help students build up creative mastery, and points out hypotheses that require more rigorous empirical tests. The paper highlights the complexity of the concept of “creative metacognition.” Based on this, we discuss methodological opportunities for refining measurement approaches to capture the phenomenon in a comprehensive manner, before offering a brief conclusion.

## 2. Design thinking as a practical approach to solving creative (ill-defined) problems

Design thinking is an approach to creativity education and practice that has been continuously implemented and refined since the 1950s (von Thienen et al., 2018, 2019, 2021; Auernhammer and Roth, 2021). Major roots of the approach go back to John Arnold, Professor of psychology and engineering at the M.I.T. and later at Stanford University, who brought together creativity experts like Joy Paul Guildford, Abraham Maslow and eminent creators like Buckminster Fuller to contribute their insights and approaches for purposes of practical facilitation. These experts co-lectured classes with John Arnold and contributed essays for the course manuscripts (e.g., Guilford, 1959/2016; Maslow, 1959/2016). Design thinking emerged as an integration of these different theoretical perspectives, yielding an approach that has been continuously applied and refined especially in the innovation education for engineers at Stanford University ever since.

While John Arnold already used the term “design thinking” in the 1950s, it became an official headline for training institutes only later. In 2004, Stanford University formally established a d.school to train students in design thinking, and a partner institute at Potsdam University, the HPI D-School, began to operate in 2007. Each of them trains hundreds of students per year. Ever since, a growing number of other universities around the globe have started to offer creativity education for trainees with varying academic backgrounds based on design thinking (Meinel et al., 2017; Wrigley and Straker, 2017; Meinel and Krohn, 2022).

During the period between the 1950s and the establishment of d.school in 2004, the theories and practices of design thinking evolved continuously, particularly through the *Joint Program of Design* initiated at Stanford University by John Arnold, Robert McKim and further colleagues. The program was offered collaboratively by the Engineering and Art departments, representing two major fields where large numbers of innovative creations emerged. However, design thinking facilitates creativity in all areas of life and is not domain-specific (Arnold, 1959/2016; von Thienen et al., 2018). Other notable areas of application include business (Brown and Katz, 2009; Liedtka and Ogilvie, 2011; Kelley and Kelley, 2013), policy (Armitage et al., 2017; Lewis et al., 2020), psychology or designing one’s own life (Roth, 2015; Burnett and Evans, 2016), among others.

In the current educational practice, design thinking trainings can take many forms, ranging from 1-h highly structured workshops to several months of self-directed project work, and in some cases even years (Carleton and Leifer, 2009; Leifer and Steinert, 2011; Meinel and Krohn, 2022). All courses provide immersive project experiences that enable students to learn through first-hand experiences (Carleton and Leifer, 2009; Kolb, 2014). A typical educational set-up at the HPI D-School in Potsdam is to form multidisciplinary teams consisting of 5–6 students, who work on a given real-life challenge or design problem. The challenge presents the creative problem space and provides the initial context for starting the creative endeavor. Often, the challenge is provided by a project partner, such as a company, a not-for-profit organization, or a Federal agency. The teams then follow a design thinking process, which starts with an exploration of the problem space before moving into the solution space. There are several process models for design thinking. One model often used at the D-School covers the phases “understand,” “observe,” “define point of view,” “ideate,” “prototype,” and “test.” When a team has gone through all phases, they can start iterating depending on the feedback they received on their solution during the testing phase. To help the team navigate through their creative process, they are usually accompanied by a design thinking coach. This person is an expert on the creative process, who supports the team by suggesting tools, methods and frameworks for each process phase. Furthermore, the coach can help to manage the team’s energy levels, moderate discussions if needed, and keep an eye on the social behavior in the group. Besides the elements of teamwork and process, design thinking education is characterized by relying on a highly variable working environment, which can flexibly be adapted to the trainees’ needs. Each team has its own team space, whether analog, virtual, or hybrid, to support different working modes and store their knowledge, such as ideas or field notes typically recorded on sticky notes.

An example of a design thinking project is described by Plattner et al. (2009). In one design challenge at Stanford University, the students were asked to build a lighting solution for countries that lacked a stable energy supply. The idea was to help children do their homework and practice reading after sunset. “The world’s largest lighting and electrical engineering companies in Europe and Korea said that such a device could not be produced for less than $ 120, more likely $ 150” (p. 15, our translation). Yet, the students set out to render such a device available for less than $ 20. Making good use of their multi-disciplinary expertise helped the students succeed.

*The physician advised the electrical engineer which lamps, which LED luminaires are (…) the most suitable for the eye while consuming as little energy as possible. The electrical engineer organized appropriate rechargeable batteries and purchased the solar panel inexpensively via the Internet. The software student described the charging procedures to store the energy in the required format on the battery (…). The business student went to New York and negotiated with the World Bank to obtain the money for a large-scale field experiment. The mechanical engineer negotiated with India via the Internet, where the outer shape of the lamp could be cast from plastic. And the sociologist flew to Mexico and South Africa to set up field trials.*

*The project was successfully completed. Several thousand lamps were built and tested by the students in India, Mexico, and South Africa. Today, these lamps are available for purchase.*

*(Plattner et al., 2009, p. 15f., our translation)*

Having considered a design thinking project example, we can now delve into its theoretical foundations and educational practices. The theoretical basis of the approach equips creators with tools to understand, observe, predict and facilitate creativity and innovation in all its forms. It is a general approach, addressing low levels of creativity and incremental innovation as well as high-level creativity and radical innovation (Arnold, 1959/2016; McKim, 1972; von Thienen et al., 2018).

By comparison, the implementation of design thinking in educational practice is geared toward the high-end spectrum of creative achievement. David Kelley, founding director of Stanford‘s d.school, describes design thinking as “a method for how to come up with ideas. These are not just ideas, but *breakthrough* ideas that are *new to the world*” (Kelley and Camacho, 2016, p. 88, emphasis added). Similarly, in a Springer book series dedicated to design thinking research, Christoph Meinel and Larry Leifer posit: “We believe great innovators and leaders need to be great design thinkers. We believe design thinking is a catalyst for innovation and bringing *new things into the world* (…) that lead to *breakthroughs*” (2011, p. xiii, emphasis added). What is characteristic in these descriptions is that on both dimensions of creative performance, novelty and value, design thinking aims for highest levels of achievement (i.e., Big-C). Solutions shall not be new to a particular audience group only, but “new to the world.” Furthermore, solutions shall not make minor incremental steps of progress only, but the approach is aiming for “breakthrough” solutions.

Overall, design thinking is an approach to solving wicked, ill-defined problems (Kahn, 1962; Buchanan, 1992) – or, as John Arnold would say, it is an approach to solving *creative problems* (cf. Arnold, 1959/2016; von Thienen et al., 2018). As a defining characteristic, “creative problems have a complete spectrum of possible solutions” (Arnold, 1959/2016, p. 77). When one is developing or evaluating those solutions, the spectrum from good to bad “is never completed. No matter how poor the worst solution existing in the spectrum is, a still worse one can be found; and in the same manner, but perhaps with more effort, a still better solution than the best one existing can be found” (p. 65). As a second characteristic of creative problems, Arnold mentions that they can be stated or defined by choosing from “an almost infinite number of concepts” (ibid.).

To further clarify the positioning of design thinking in terms of creativity concepts and creative problem-solving, a comparison can be drawn to the works of Alex Osborn, who also developed theories and practices during the times when design thinking emerged. Osborn’s works were well-known to John Arnold, and extensively incorporated into his works (1959/2016), albeit not uncritically. Osborn (1953) is widely recognized for his brainstorming methodology, which has become part of the design thinking toolbox (d.school, 2010). In the process of incorporation, Arnold acknowledged “the usefulness of brainstorming in the idea-getting stage” (1959/2016, p. 108), meaning that it can be a helpful method in a specific phase of the creative process when creators begin to explore the solution space.

In terms of creative problem-solving, Osborn defines:

*The methodology of creative problem-solving usually includes some, or all, of these procedures:*

*Orientation: Picking out and pointing up the problem.*

*Preparation: Gathering the data.*

*Analysis: Breaking down the relevant material.*

*Ideation: Thinking up ideas by way of possible solutions.*

*Incubation: Letting up, in order to invite illumination.*

*Synthesis: Putting the pieces together.*

*Evaluation: Verifying the tentative solutions.*

*(Osborn, 1958, p. 23)*

The design thinking approach to creative problem solving shares many similarities with Osborn’s model, in that it describes a comprehensive process that begins well before and extends well beyond the ideation stage. However, design thinking employs different terminology and may not emphasize certain aspects as much. For example, design thinking pioneer McKim (1972) extensively discussed the notion of incubation and the importance of unconscious information processing, while these factors play a more limited role in newer method compilations (d.school, 2010).

**FIGURE 1:**
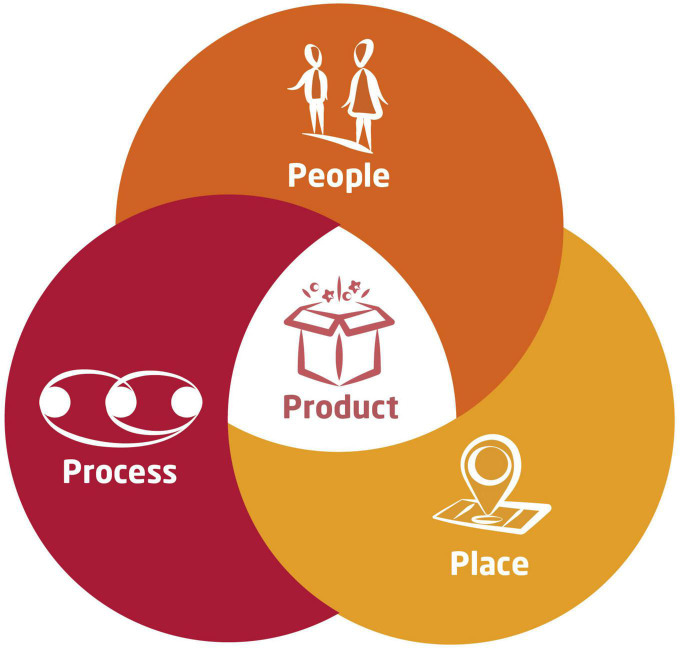
Design thinking provides knowledge and training in four domains (4P) to facilitate creativity and worthwhile innovation. These four domains are: creative *P*roducts, *P*rocesses, *P*eople, and *P*laces.

Another way to position design thinking among related works can be by reference to a framework suggested by Rhodes (1961), often referred to as the 4P account of creativity. It reviews factors that are relevant to creativity in four domains: creative Persons, Process, Press and Products. Design thinking began to take shape already before this model was published, and then it developed somewhat independently. Design thinking arrived at its own 4P framework, highly similar to Rhodes’ headlines and yet different in nuances. The 4P framework used in design thinking provides resources and training for students in the domains of creative ***P***roducts, ***P***rocesses, ***P***eople, and ***P***laces (Figure 1). Subsequently, the paper reviews the role of metacognition across these four domains of creativity expertise. We submit that these areas are of paramount importance for metacognition as a means of enhancing creative performance. Comprehensive measures of creative metacognition can be obtained by screening each of these domains separately.

Notably, this paper proposes four domains of creative metacognition, while the widely endorsed definition by Kaufman and Beghetto (2013) distinguishes only two (cf. Section “1. Introduction”). Is the four-domain model of metacognition in design thinking simply a different way of organizing the content of “self-knowledge” and “contextual knowledge” described by Kaufman and Beghetto, or does it introduce new elements? In many aspects, it will be a reorganization, although some new facets are also brought to light. Compared to the two-factor model, the four domains provide a more systematic framework for understanding the various types of metacognition involved in creative thinking, which can aid comprehensive assessments of competency. While we will define metacognition in the four domains below (in Sections “3. Creative metacognition on products,” “4. Creative metacognition on processes,” “5. Creative metacognition on people,” and “6. Creative metacognition on places”), we would like to highlight some subtle distinctions to assist with orientation:

Metacognition related to products is concerned with problem characteristics and resulting problem-solutions, i.e., creative products. Thinking about creative products is only indirectly related to thinking about oneself (self-knowledge) or thinking about the context of one’s creative work (contextual knowledge). The search for a particular product or problem-solution is the reason and purpose of creative work. Furthermore, the creative product provides a central reference point for determining creative achievements (cf. Section “1. Introduction”). Against this background, we argue that the creative product – and metacognition on it – is a highly important category in the analysis of creative metacognition. Therefore, we believe it should be directly addressed, as suggested in the 4P approach, rather than being treated merely as a side detail in the two-factor model of self-knowledge and contextual knowledge.

Metacognition on processes involves monitoring the strengths and weaknesses of the activities and strategies used during a creative process. This factor is similar to “contextual knowledge” of how to be creative in the two-factor definition. In design thinking, the focus lies on how to be creative in the most effective way possible, by providing guidance on different phases in the creative process and suggesting actionable next steps, such as general strategies or specific activities.

Metacognition on people involves monitoring the strengths and weaknesses of individuals and groups involved in creative activity, and their contributions to the overall creative outcomes. This factor goes beyond self-knowledge, because it involves not only an awareness of one’s own creative strengths and limitations, but also an understanding of how others co-shape the creative process and its results. The involvement of others in metacognition on people is not strictly “contextual knowledge” as described in the two-factor definition, since collaborators in a project do not just provide the “context” or background for the individual creator, but instead the creative team and even larger communities can be a significant unit of creative activity on their own (Roth, 2015; Weinberg, 2015; Reiter-Palmon, 2017).

Metacognition on places involves monitoring the strengths and weaknesses of past, present, and future environments for the creative project and for living creatures in general. This factor is linked to contextual knowledge on where to be creative. It also involves a comprehensive assessment of how physical and social environments support or inhibit creativity, and a call to reshape environments for improved creative performance. Furthermore, beyond questions of what motivates creative action, place metacognition involves reflections on how creative outcomes impact the world at large, such as their effects on diverse species.

In addition to these conceptual clarifications, implications discussed later in this paper suggest that the four domains of metacognition identified in design thinking extend beyond the two-factor model of self-knowledge and contextual knowledge. Preliminary evidence indicates that metacognition on people and processes may develop earlier than metacognition on products and places (Section “8. Future research suggestions”). Therefore, if metacognition across the four domains matures at different points in time, it can be practical to keep them separate. Moreover, the four-domain model of metacognition calls for a different methodological approach to measuring creative metacognition in the service of more comprehensive and systematic assessments (Section “9. Next steps in the measurement of creative metacognition: from accuracy scores to more comprehensive assessments”). According to this framework, accuracy scores alone are insufficient, and additional methods are needed to capture the complex phenomenon of creative metacognition in a valid way.

## 3. Creative metacognition on products

*Creative metacognition on products involves the monitoring and control of problem and solution characteristics in a creative project. It focuses on the strengths and weaknesses of creative project goals – i.e., the products creators try to develop – and accordingly on the strengths and weaknesses of solutions created to date.* An example of product-related cognition is when a creator endeavors to sketch decorations on a birthday card and perceives their current embellishments as inadequate, because they fail to cover large blank areas still left on the card. Through metacognitive reflection, the creator may come to realize that the initially endorsed task of randomly filling blank spaces with decorations is not suggestive of high-quality creative results. *Creative metacognition on products enhances creative performance by guiding creators toward ambitious projects and respective solutions in terms of product novelty and value, ultimately leading to higher-quality creative outcomes.*

**FIGURE 2:**
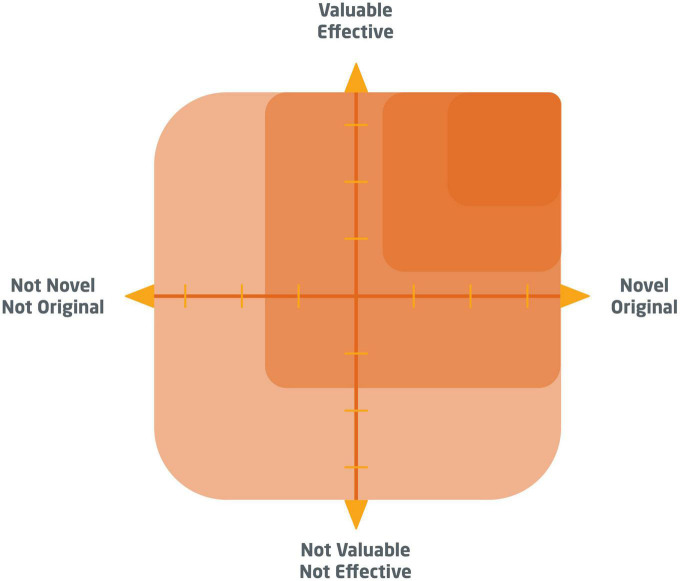
Creators achieve high levels of creative performance when their final work outcome is both highly novel and highly valuable. Product metacognition monitors novelty and value not only for final work outcomes but also for likely-to-emerge-products at any time during the creative process. One important assessment basis can be the task statement (work goals) and evaluation criteria (success metrics) used in a project.

The goal of creative work is to develop a *new* and *valuable* solution to some problem (Runco and Jaeger, 2012; Paul and Kaufman, 2014; Royalty and Roth, 2016). The creative product can be tangible or intangible, such as a technology, painting, piece of furniture, service, theory, song, or any other novel and valuable outcome (Arnold, 1959/2016; Paul and Kaufman, 2014). Creative metacognition on products assesses novelty and value in existing products, but also in likely-to-emerge products in the creator’s project. It is concerned with problem characteristics (such as project goals, task statements, evaluation criteria, success metrics) and solution characteristics (such as the novelty, value, and fulfillment of task constraints among ideas, prototypes, or final work outcomes). In order to enhance creative performance, creators need to strive for highly novel and highly valuable work outcomes (Figure 2).

Notably, creative products only emerge by the end of a successful process (Corazza, 2016; Corazza and von Thienen, 2021). From the beginning of a project, metacognition on products can guide creators toward high-quality outcomes by taking into consideration the relationship between given problems and emerging solutions. It is important to note that creators make decisions regarding final product characteristics, including product novelty and value, based on the tasks they set for themselves. Therefore, creators must continuously monitor and reflect on the type of outcome they aim to develop. Although these reflections occur throughout the creative process, they are examples of product metacognition as they relate to (emerging) products. Ultimately, metacognition across all four domains is interconnected.

One of the key understandings that students acquire in design thinking education is that the task they endorse plays a significant role in determining their final creative outcome. Trainees often hear that their statement of the project goal should be a sentence that no other human has said or thought before (cf. hypothesis 1 out of 15 in section 8). Unconventional task statements guide the creator toward terrain that has not yet been explored by humanity, aiding in the search for a solution that would be new to the world. However, problem statements should not just be unconventional in random ways. Instead, they should be grounded in a deep analysis and insights on the subject matter, focusing on important problems or basic needs that humanity has to address. Finding a solution to a problem framed in such ways implies a breakthrough.

A historical example of this approach is provided by Arnold (1959/2016). For the sake of clarity, we will outline his procedure, which involves both cognition and metacognition in relation to the creative process and the ensuing creative products. Having introduced the case example, we will single out specifically metacognition on products.

Arnold sets out with the overarching objective of redesigning mobility. An initial exploration of the problem space points to a range of issues including “Immature Drivers[,] Traffic Deaths[,] Congested Cities[,] (…) Bad Traffic Regulations [and] Inadequate Highways” (p. 94). Each of these issues could become the main target of an emerging creative project. In light of the identified issues, Arnold formulates a first, tentative problem statement: “MAN MUST BE KEPT MOBILE – YET NOT BE OVERLY FRUSTRATED & IN CONSTANT DANGER OF SUDDEN DEATH” (ibid.). However, this statement still seems to be scratching the surface rather than representing a deep analysis that offers a new, unique, and intriguing perspective on the subject. Asking himself why people need to be mobile, Arnold answers that often the underlying need is communication. People drive to another place in order to communicate with other people, e.g., at work, or to reach machines with which they want to interact, such as coin-operated laundry machines or pay phones. This analysis allows him to iterate and reformulate the problem statement as follows: “MAN MUST BE ABLE TO COMMUNICATE FREELY WITH OTHER MEN AND MACHINES” (ibid.). Based on this vision, he ponders several more concrete projects to choose from, each entailing its own types of creative outcomes: “(i) How can we better use public transportation – can subways be used to carry freight and keep some trucks out of city? (…) (ii) What means of communication can we substitute for face-to-face meetings (e.g., closed circuit TV)? (iii) What are some of the wildest approaches (e.g., disposable cars)?” (ibid).

In order to choose between different tasks, the creator can evaluate likely-to-emerge products. Arnold’s refined task statements suggest subways that carry freight, solutions for remote work inspired by closed circuit TVs, or radical solutions like disposable cars. These solutions can be assessed for their novelty and value. While subways already exist, using them to carry freight and keep trucks out of the city could be practically beneficial, but may not be radically novel. In contrast, wild approaches like disposable cars would be very novel, but their practical value is uncertain. On the other hand, reducing mobility through remote work solutions based on closed circuit TV seems promising in terms of both novelty and value. Indeed, this proposal was extremely visionary in the 1950s. It anticipated discussions on the benefits of remote work as an avenue to reducing mobility – an issue that became increasingly important in light of the environmental movement (Ong et al., 2014). In this sense, Arnold’s approach was ahead of its time by half a century.

In this project example, Arnold initially formulates a broad problem statement and later converges on more unique and specific project goals. Metacognition on products leads to the revision of the first problem statement. Arnold begins with a relatively common statement about people needing to be mobile without frustration, with associated evaluation criteria such as metrics of city congestion. However, this task specification can be fulfilled by many standard solutions like building yet another highway – approaches that are not particularly novel or valuable in the grand scheme of things (cf. Figure 2). Therefore, the task statement does not propel the creator into novel, unexplored, and yet promising terrain. Metacognition on products reveals these weaknesses and prompts the creator to rethink their goal. By iterating forward toward a more unique and promising outcome vision, Arnold arrives at the revised task statement of reducing the number of occasions where people would need to be mobile. This new project goal shows creative potential, as it points to solutions that are significantly novel and valuable, including remote work approaches via tele-conferences as a means to reduce traffic—thought up in the 1950s.

A modern example of how to rephrase tasks in ways that can lead to more highly creative outcomes, is provided by Kelley and Kelley (2013).

*For example, in retail environments, we’ve discovered that if you change the question from “how might we reduce customer waiting time?” to “how might we reduce perceived waiting time?” it opens up whole new avenues of possibility, like using a video display wall to provide an entertaining distraction.*

*(Kelley and Kelley, 2013, p. 23)*

In this example, the project goal is again refined iteratively. The initial task of reducing customer waiting time can be accomplished by a number of already common solutions, such as adding more check-outs in the store or refining details in the checkout system. Success metrics would likely include average checkout times or the maximum wait time during peak hours. However, this task description and associated evaluation criteria are not particularly innovative. A more radically novel and potentially valuable solution might be achieved by flipping the goal on its head: maximizing the time customers want to spend at the checkout by making the process intrinsically enjoyable.

In summary, product metacognition helps to ensure that products satisfying task constraints are both highly novel and highly valuable. To enhance creative performance, it is crucial to optimize both the creative task, and the products within the solution space created by that task, in ways that maximize the novelty and value of work outcomes.

## 4. Creative metacognition on processes

*Creative metacognition on processes involves the monitoring and control of activities and strategies used during the creative process, with the aim of optimizing them for the best possible creative outcome.* An example of process-related cognition is a student who begins a particular task they are given by deciding on a method they want to try in order to solve the challenge. By means of metacognitive reflection, the student recognizes that their work process begins with an exploration of the solution space, skipping a phase of exploring the problem space, which could be a means to enhance the creative potential of the overall project. *Creative metacognition on processes enhances creative performance by helping creators determine strengths and weaknesses in the activities or strategies used by creators – both themselves and others – and by optimizing approaches in order to arrive at the best possible creative outcomes.*

**FIGURE 3:**
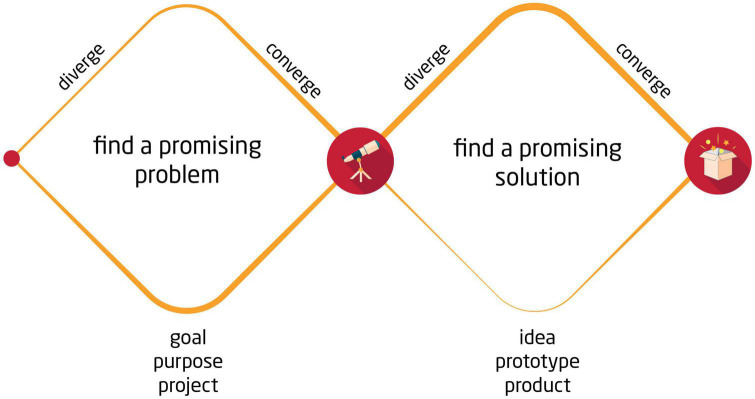
The design thinking methodology starts with an exploration of the problem space, resulting in the formulation of a specific problem statement that outlines the desired outcome of the project. Based on this clarified vision, the design thinking process shifts to the exploration of the solution space. The final goal is to identify one or more novel and valuable solutions, as outlined in the project vision.

As in the Double Diamond process models that have been set forth by the British Design Council from 2005 onward (Design Council, 2005), design thinking students learn to carefully explore problem spaces, from which possible tasks can be selected, and only afterward explore solution spaces that result from accepting a particular task (Figure 3). Process metacognition involves an awareness of one’s current position in the creative process, such as whether one is operating in the problem or solution space, and which specific process phase one is in. For instance, the phases of ideation versus testing prototypes are both means to explore the solution space, but they occur at different points in time and involve different objectives or recommended strategies. As the d.school (2010) method compilation suggests: “Be Mindful Of Process[:] Know where you are in the design process, what methods to use in that stage, and what your goals are” (p. 3). Therefore, process metacognition also involves reflecting on appropriate actions to take. For example, students learn not to generate solution ideas while exploring the problem space, but to postpone this step until a later stage. Similarly, students learn to refrain from critical judgments during the ideation phase when generating solution ideas, instead deferring critical evaluations until the subsequent stage of testing prototypes (McKim, 1972; d.school, 2010).

In terms of a general heuristic, design thinking trainees learn to consider diverse perspectives on the problems they tackle (Plattner et al., 2009; d.school, 2010). For instance, in creative projects with business partners, this may include interviewing the target user group, investigating the views and experiences of “extreme users,” such as those who are opposed to or highly enthusiastic about a product, as well as other relevant parties like retailers and delivery services. By incorporating multiple views beyond the traditional “expert” perspective (such as that of the company manager), creators can gain a more nuanced understanding of potential project goals and resulting products, enabling improved outcomes compared to more conventional work approaches that operate within the confines of a particular discipline or accept the perspective of a select authority.

Design thinking students are also taught to critically question the inputs from various stakeholders. This means to look out for contradictions, conflicts of interest, or evaluation criteria that clash with a healthy satisfaction of basic human needs (d.school, 2010). One classic example from early design thinking literature bears on the US automobile industry of the 1950s (McKim, 1959/2016; von Thienen et al., 2019). In the design process, the professionals involved developed and selected low, sportive automobile looks, because these were in high demand with the customers. However, in doing so, car manufacturers accepted seat designs that put car users into unhealthy sitting positions. The designers accepted this knowingly, letting themselves be guided by marketing studies on consumer choices. However, from the design thinking perspective, better designs could be produced by resolving the conflict, developing novel car models that would be both attractive to the customers in terms of form, but also healthy to sit in. Overall, based on considerations of the affected basic human needs, creators can detect deficiencies in prevailing solution approaches, ultimately to create better solutions for the future (von Thienen et al., 2022; Borchart et al., 2023).

Importantly, design thinking students learn to work beyond one approach that is all too common in education, where students (shall) try to internalize the judgments of their teachers and emulate experts. Arnold (1956) calls this the authority approach to problem solving and describes what happens as follows: “You go and ask the grand old man the answer to your problem. He tells you, and then you accept that without question. (…) This procedure accepts the established answer as the right answer. Even though there may be a great multiplicity of better answers” (p. 4). Design thinking is certainly not opposed to learning the views of domain experts. Indeed, design thinking is all about learning as quickly as possible (Leifer and Steinert, 2011) – but from more than one perspective. High-level creativity requires thinking beyond the already established views of recognized experts [cf. Sections “1. Introduction” and “2. Design thinking as a practical approach to solving creative (ill-defined) problems”]. To ensure that students are not tempted to endorse prevailing expert views uncritically, design thinking brings together a great diversity of viewpoints in the classroom. Not only do the students come together in interdisciplinary teams, but also the teaching team is compiled in such a way as to encompass a diversity of perspectives. “In the d.school, all classes must be team-taught” (Roth, 2015, p. 150). Ideally, teachers represent diverse approaches, so that the students do not get trained in a single paradigm and follow it blindly. Teachers are welcome to critically reflect on each other’s approaches and have controversial exchange. “The sharing of sensibilities and different points of view enrich the educational experience for students and for teachers, and this occurs when we bring teachers from different backgrounds into the same classroom” (p. 152).

Another strategy deployed in design thinking to ensure high-quality outcomes is an iterative work approach, following the *ETC* model (McKim, 1972). The acronym stands for *E*xpress (your solution ideas), *T*est (your solution ideas) and *C*ycle.

*Once you have expressed a number of ideas (…), you are ready to evaluate them. Judgment, deferred in the Express phase of ETC, is fully exercised in the Test phase. Now is the time to be self-critical, not before (…).*

*Testing, of course, implies criteria. In the early rounds of ETC, criteria are usually imprecise, incomplete, and implicit. Initial criteria are also frequently inaccurate. The final function of the Test phase is to review criteria and to state them more exactly. (…) As you formulate and refine your criteria, record them in writing. The revised statement of criteria is an invaluable aid in the next round of ETC.*

*(McKim, 1972, p. 121)*

Such an iterative work approach is used in design thinking, no matter what specific process model the creators follow.

## 5. Creative metacognition on people

*Creative metacognition on people monitors the strengths and weaknesses of people in a creative project. It regulates their personal development, as well as the development of small teams or larger creative communities, to achieve the best possible creative outcomes*. An example of people-related cognition is a student who works on a creative project alone. Upon metacognitive reflection, the student becomes aware that their envisioned solution requires IT skills that the person does not have and cannot learn in a short amount of time. However, another student in the dormitory has exactly these competences and might be interested in teaming up. *Creative metacognition on people enhances creative performance by helping creators develop their own creative strengths and overcome weaknesses, and by helping to optimize the involvement of diverse people in the creative project, in order to arrive at best possible creative results.*

**FIGURE 4:**
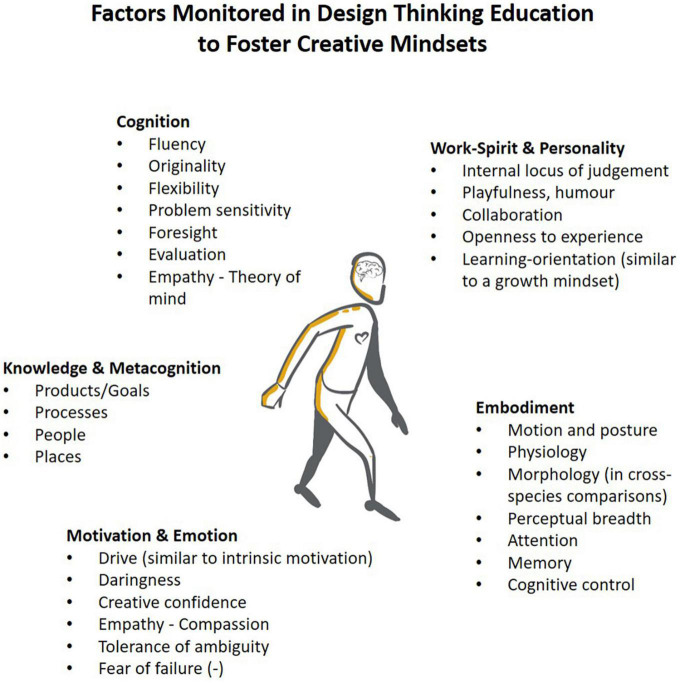
Design thinking involves monitoring and developing various factors related to people, often referred to as the “creative mindset”. These factors serve as the focus of coaching interventions in design thinking education and as targets for measurement in research.

As can be expected from the origins of design thinking, based on the contributions of people like Guilford and Maslow, a strong sensitivity to characteristics of a creative mindset informs the approach. Figure 4 provides an overview of factors that are typically monitored in design thinking, serving as a basis for interventions by the design thinking coach in educational practice, providing training aims, and parameters to quantify in design thinking research.

Due to the early formation of the approach, some design thinking terminology differs from concepts that later became prominent in creativity studies. For instance, “drive” refers to the emotional energy and enthusiasm with which creators pursue their projects, especially when facing hardships (Arnold, 1959/2016; von Thienen et al., 2018). Much of this factor later came to be discussed as “intrinsic motivation” in psychological research (Deci, 1972), while “drive” is also closely related to behavioral aspects of perseverance in the face of obstacles and emotional aspects of excitement about one’s creative project.

Any of the factors listed in Figure 4 can become a target of metacognitive reflection among trained creators. For instance, creators may recognize that they do not display much drive regarding the project they are currently tackling: They are not particularly excited about it. They know that enthusiasm for their work is essential to deliver the best they can in ambitious creative work (Arnold, 1959/2016; McKim, 1972). Therefore, adapting the project goal seems recommendable if high levels of creative performance, and maybe even breakthrough outcomes, are desired.

While there are too many factors to review them individually in relation to creative metacognition, we want to address the following examples: flexibility, empathy including theory of mind, an internal locus of judgment and daringness, as well as collaboration.

In the *Alternative Uses* task (Guilford, 1956), a standard test in creativity research, *flexibility* is usually evaluated by assessing the conceptual diversity of the examinee’s ideas. While this kind of ideational flexibility is monitored closely in design thinking as well, other forms of flexibility, such as thinking strategies, are considered crucial as well. Design thinking education highlights the use of diverse strategies, such as mathematical vs. visual vs. verbal thinking (McKim, 1972; Adams, 1974; von Thienen et al., 2021) to empower creators in their choice of relevant project goals, and to help with overcoming obstacles in case an initially chosen approach fails to yield the desired solution.

Another factor of interest is *empathy*. Some design thinking process models begin with a work phase called “empathy” (Plank et al., 2021), where creators talk to representatives of diverse stakeholder groups and make behavior observations to better understand their perspectives. Design thinking typically involves “a human centered perspective, where innovators build empathy with users” (Verganti et al., 2019, p. 1). Overall, the approach recognizes that no single expert group has the authority to determine the ideal solution or product for everyone. Instead, it acknowledges that different people may have varying interests, and that creators should be mindful of this diversity when choosing their project goals and success metrics. For example, in the context of car manufacturing, conflicting ideals such as maximizing horsepower versus sustainability can result in diverse solution preferences (Hula et al., 2022). Creators must be aware of how their proposals may be perceived differently by different audiences.

From a research perspective, empathy is a two-fold phenomenon (Plank et al., 2021), involving emotional components (e.g., “compassion”) and cognitive components (“theory of mind”). When creators use *theory of mind*, they develop beliefs about other people’s emotions (such as “my teacher will love this idea of mine”) and thoughts (such as “the teacher will think that my solution is ideal because it is so cheap and lightweight”). These beliefs can be right or wrong. From the perspective of creative metacognition, having a high level of accuracy in theory of mind is desirable, as it enables creators to make informed decisions and predict idea evaluations of others.

While understanding the perspective of others is a very helpful basis for creative work, creators need not take sides with any particular stakeholder group. Ultimately, the creator is responsible for their goals. They need to maintain an *internal locus of judgment*, meaning that they introduce their own evaluation criteria in a project based on their best knowledge and conscience of what would be desirable in the field (Arnold, 1959/2016). This responsibility cannot be deferred to teachers or other experts. Furthermore, “[t]he creative person has to be *daring*. He has to be a leader in his group for society, and he must constantly take calculated risks in his attempt to find better solutions to the problems that face mankind” (p. 87, emphasis added). In a number of historic cases of Big-C achievements, the creators set themselves goals that seemed unattainable, as “reason and analysis (the experts) said that it couldn’t be done” (p. 104). However, the creators decided to move ahead with their daring visions and ultimately managed to develop breakthrough solutions. Setting aside expert opinions can be a strategic choice and calculated risk in the exploration of novel territory, on the lookout for breakthrough solutions. From a creative metacognition perspective, design thinking teaches creators to seek a thorough understanding of diverse perspectives including the views of recognized experts, but ultimately creators have to make their own informed choices.

Finally, *collaboration* is a crucial aspect to consider in design thinking (Carleton and Leifer, 2009; Plattner et al., 2009; Leifer and Steinert, 2011; Roth, 2015; Weinberg, 2015). It starts with acknowledging that many great creations are made possible by building on the works of previous generations (Dean et al., 2014; von Thienen et al., 2023b). For example, the invention of the car would not have been possible without prior inventions of the wheel and the motor. The implicit collaboration with prior creators allows for sophisticated developments that would not be possible if creators had to start from scratch, for instance with the intellectual and material resources of the Stone Age (Dean et al., 2014; Corazza and Glãveanu, 2020; von Thienen et al., 2023b). In design thinking, trainees learn to recognize this through mottos such as “All Design Is Re-Design” (Meinel and Leifer, 2011, p. xv). Design thinkers familiarize themselves with past solutions to similar problems and draw inspiration from various fields facing analogous issues (d.school, 2010). In addition, direct collaboration is important, as design thinking usually takes place in a network of social support systems (Carleton and Leifer, 2009). Trainees work in interdisciplinary teams, receiving topic-related input and feedback from project partners, while receiving creativity-related input and support from design thinking coaches. Trainees also exchange experiences and work with stakeholders such as potential users or field experts (Plattner et al., 2009; d.school, 2010). Acquiring knowledge through collaboration is considered just as important as learning to master topics on one’s own. Trainees learn to screen their project for missing information and expertise, closing gaps and increasing the project’s overall potential in a highly collaborative spirit, by readily reaching out to others and connecting with them. Innovation is truly a team sport.

## 6. Creative metacognition on places

*Creative metacognition on places monitors the strengths and weaknesses of past, present and future places both as environments for creative projects and for living creatures in general. It involves the control and shaping of work environments to enhance creative performance, as well as the control and shaping of creativity to maintain a livable planet.* An example of place-related cognition is the decision of an IT-developer to move from one PC to another in order to improve the working conditions in the ongoing project. By means of metacognitive reflection, the creator may recognize that many more options exist for potentially favorable place changes. For instance, by leaving the solitary office and instead going to the Cafeteria, there would be more opportunities to meet people, with whom the general solution approach might be discussed. On a larger scale, by moving from one country to another, the funding opportunities for the project could be improved. *Creative metacognition on places enhances creative performance by helping creators select and shape environments that facilitate creativity. Additionally, it prompts creators to constrain themselves and to advance understandings of creativity in ways that contribute to a livable world.*

The final pillar to consider in the 4P design thinking model is “Place.” It holds significance for creative work in two crucial ways: Firstly, the environment can facilitate or obstruct creative work (Atchley et al., 2012; von Thienen et al., 2012; Guegan et al., 2017; Bourgeois-Bougrine et al., 2020). Creators need to recognize these impacts and take corrective actions if desired. Secondly, the solutions devised by creators will shape the future environment of people and other organisms (Artifact Group, 2023; Borchart et al., 2023; von Thienen et al., 2023b). Therefore, it is imperative that creators anticipate the impact their work may have in the future, in order to take better decisions today.

The impact of surroundings on creativity and innovation is widely recognized. The immediate environment in which creative activity takes place has a discernible effect, as in a particular room, a building, or in nature (Atchley et al., 2012; von Thienen et al., 2012; Guegan et al., 2017; Bourgeois-Bougrine et al., 2020). Furthermore, broader contexts like geographic regions or political climates can also play a crucial role in influencing creativity and innovation (Weiner, 2016; von Thienen et al., 2023a).

Overall, places encompass any environment that influences the feelings and activities of individuals or groups, including their creative inclinations (von Thienen et al., 2023b). Some places are clearly defined by physical boundaries, such as an office space in a building. Other places are socially defined, like a “zone of psychological safety” characterized by trust among colleagues, or a “rigid family” that restricts creative freedom among its members. Additionally, places can be physical, digital or a combination of both. They can range from small, such as a computer desktop background, to large, such as a country, continent, or planet during a specific period in time.

Design thinking emphasizes the importance of considering the impact of environments on creativity. It encourages the use of interventions, such as modifying surroundings, to optimize creative potential (Klooker et al., 2019). For instance, an organizational environment where failure is not tolerated can restrict creative work (Kelley and Kelley, 2013). Thus, creators are encouraged to explore ways to establish a more conducive and supportive atmosphere for creativity. In design thinking education, trainees are taught to habitually alter their surroundings to facilitate tasks, supporting creative efforts through appropriate environmental changes. In the training facilities, furniture such as couches, tables, and whiteboards are equipped with wheels, allowing for easy rearrangement (Leifer and Steinert, 2011; Doorley and Witthoft, 2012). Through practice, students learn to create different setups for various purposes in the course of the creative process.

The second way in which places are important lies in the impact of the products created. In design thinking classes, one exercise asks students to identify an item in their immediate environment that has not been shaped by human creativity, something like a “piece of untouched nature” (von Thienen et al., 2023b). This usually proves to be difficult. Nearly everything that humans surround themselves with, from technology to houses, furniture, books, paintings, music, and more, is the result of someone’s creative process. Even the raw food that humans eat, like apples and salad, is the result of a food production process that has been thoroughly shaped by human creativity. It builds on the invention of agriculture in prehistory and also relies on recent developments, such as the invention of tractors, breeding techniques, fertilizers, insecticides, and efficient delivery chains, as well as policy-making through agricultural legislation and trade law. When creators develop new solutions, they shape the places that everybody might be living in tomorrow (Meinel and von Thienen, 2022). Humans surpass other species in transforming the world to meet their needs, which has significant implications for all life on earth. This has led to debates about labeling our current geological epoch as the “Anthropocene,” an era marked by human activities that affect life conditions globally (Zalasiewicz et al., 2011). In case creators succeed with ambitious projects, their processes result in new solutions that become part of the world and shape it, potentially even on a global scale, which happens for better or worse (cf. Artifact Group, 2023; Borchart et al., 2023). Engaging in metacognitive reflections on the impact of their creations can help creators improve their project goals and avoid causing harm. Such reflections can also guide them in deciding when to refrain from pursuing certain creative ambitions (McKim, 1959/2016; Kaufman and Beghetto, 2013), as the intended solution might be causing more harm than good in the grand scheme of things.

## 7. Strategies of educating for creative metacognition in design thinking

The development of metacognition in creativity education takes time for several reasons. In particular, the human working memory is limited, and trainees need their mental processing capacity to master creative cognition before they can also learn to master creative metacognition in comprehensive ways (Martinez, 2006). Furthermore, there is a need for a robust knowledge base, for instance in each of the 4P domains.

Design thinking facilitates the development of creative metacognition using three major strategies. All strategies are implemented by giving students the opportunity to practice this mental capacity, and by placing them in situations that gradually require more and more metacognitive reflection and self-management. The first two strategies build on a distribution of cognition versus metacognition; the third strategy makes thinking tangible. In terms of future research perspectives (Section “8. Future research suggestions”), it can be hypothesized that each of the three strategies leads to an increase in creative metacognition competencies among students.

A first strategy is to separate cognition from metacognition in terms of time and space. In design thinking, this is achieved by allocating specific times for active work during which students engage in their projects (creative cognition), as opposed to reflective debrief sessions that take place by the end of their work days (creative metacognition). The reflective sessions provide an opportunity for students to share their perceptions, reflect on experiences, and develop a mutual understanding of what worked well and areas for improvement. Practical instructions on the hosting of debrief sessions can be found in the d.school *Facilitator’s Guide*^1^ and the *Design Dash*.^2^ A sample method used for debriefing is *I like, I wish* (d.school, 2010, p. 48), where creators reflect on what they enjoyed about a particular creative project experience and what they would wish for in future or similar situations. The physical environment also plays a role in the distinction between cognitive modes, with high tables and stools used for active work, versus low chairs and couches for relaxed conversations in debrief sessions (Doorley and Witthoft, 2012). This approach highlights the role of tools, time-boxing and supportive room setups in the development of creative metacognition.

The second strategy distributes cognition versus metacognition across people. In the literature, this approach is discussed under the headline of “person-plus” thinking (Perkins, 1993; King, 1998). In design thinking, each student team is usually accompanied by a coach. As a design thinking expert, the coach facilitates process-related decisions, suggests methods, monitors the teams’ mood, energy, social dynamics as well as project progress, and provides a role model for creative metacognition along the way. Ideally, coaches only intervene when necessary. They help students overcome obstacles, maximize their learning outcomes in the project, and support students in realizing their creative potential. In this way, the student team can focus on the content of their project, apply the suggested creative methods and exercise creative cognition. As students progress in their studies, they take more responsibility for structuring their own process, and thus become more responsible for creative cognition and metacognition alike. This approach highlights the use of distinct roles, social support systems, and role models in the development of creative metacognition (cf. Vygotsky and Cole, 1978; Bandura, 1997; Carleton and Leifer, 2009).

In terms of a third strategy, the development of metacognition can be facilitated when thinking becomes manifested or observable in some way, providing obvious content for reflection (Martinez, 2006). This is the case in design thinking education for several reasons. Firstly, students usually work on creative projects in teams, resulting in a largely verbalized creative thinking process. In continuous ways, students articulate their thoughts, such as: “Let’s do A,” “That has advantage 1,” “It would be good to have advantage 2 as well,” “Then let’s do B.” Secondly, teams use prototyping materials to create tangible representations of different problem views and solution ideas, and keep written notes (McKim, 1972; Dow et al., 2009; Edelman and Currano, 2011; Leifer and Steinert, 2011). Because thinking is explicit and takes tangible form, it becomes readily available content for monitoring and reflection. Lastly, design thinking teams are composed of multidisciplinary members with diverse academic and cultural backgrounds whenever possible. Through their diverse perspectives, team members invoke different thinking styles when working together on a creative project (cf. Section “4. Creative metacognition on processes”). To understand each other, they often engage in mutual questioning and explanation of how they think and why they take these approaches, resulting in a natural interplay between creative cognition and metacognition. Overall, this approach highlights the role of explicit thinking and of tangible representations of thoughts in the development of metacognition.

## 8. Future research suggestions

So far, design thinking theories and practices have been introduced. This can help in structuring important components of creative metacognition, which presents itself as a complex concept in professional practice and education. Yet, when it comes to observing empirical relationships, much work still needs to be done. This section begins by highlighting some research areas that warrant further attention. Subsequently, Section “9. Next steps in the measurement of creative metacognition: from accuracy scores to more comprehensive assessments” will outline recommendations for study designs and measurement approaches.

Overall, design thinking aims to help students develop creative mastery and achieve significant creative accomplishments (Section “2. Design thinking as a practical approach to solving creative (ill-defined) problems”). It imparts extensive knowledge and practical experiences in the 4P domains (Sections “3. Creative metacognition on products,” “4. Creative metacognition on processes,” “5. Creative metacognition on people,” and “6. Creative metacognition on places”), using three main strategies to facilitate the development of creative metacognition (Section “7. Strategies of educating for creative metacognition in design thinking”).

The impact of design thinking education has been widely assessed, showing rapid enhancements in creative self-efficacy (e.g., Rauth et al., 2010; Traifeh et al., 2020), improvements in objective creative performance associated with characteristic changes in people’s brain activities when working on creative tasks (e.g., Bott et al., 2014; Saggar et al., 2017), as well as long-term impacts on people’s work approaches and career trajectories (e.g., Royalty et al., 2014). Yet, these studies on the impact of design thinking education have not addressed creative metacognition so far. Indirect evidence can be seen in an experiment with 116 high school students (Noweski et al., 2012). Here, participants were randomly assigned to work on the same creative challenge, facilitated either in design-thinking fashion, or based on another educational model for project-based learning, the Dewey-Kilpatrick approach. One major difference between the two teaching approaches is a clearer distribution between cognition and metacognition in design thinking, as outlined above (Section “7. Strategies of educating for creative metacognition in design thinking”). On all dependent variables assessed in this experiment, design thinking had more beneficial effects, including the development of social skills among students, task enjoyment and student-teacher relationships. However, in the future, direct assessments are recommended to trace the development of creative metacognition over time, and to determine the impact of metacognitive competencies on resulting creative products.

Another open area for future research concerns the timing of the development of creative metacognition in the 4P areas. It is a regular observation, though not yet systematically researched, that students seem to engage in metacognitive reflections concerning people and processes already by the beginning of their training: They readily talk about their own strengths and weaknesses as creators or mention how easy or difficult it is for them to apply certain methods. In contrast, metacognitive reflections on products and places seem to emerge later.

With regard to product metacognition, John Arnold makes pertinent observations:

*Most students, for example, if you give them a problem to do, (…) jump right away into some kind of procedure on how to solve it. They don’t sit down and try to think “What am I trying to do? What is the goal I am aiming for? (…)” They start looking for some method. They try, oh, integration, or a differential equation (…).*

*I am sure that if a great deal more time were to be spent in actually formulating a (…) comprehensive picture of what you are trying to do, one would be much more effective in arriving at an outstanding solution.*

*(Arnold, 1956, p. 28)*

According to this analysis, most students initially display limited competencies in product metacognition. They are not aware that their final work outcome is likely to lack novelty and value if they do not have a unique and encompassing set of goals for the product they seek to create. As a result, they are disoriented about favorable steps to take in the process.

With regard to place metacognition, Weinberg et al. (2014) make relevant observations. According to their analyses, experienced students create their own work environments and strategically choose work locations to support different tasks throughout the creative process, whereas novice students tend to remain in one work location and make no modifications throughout the project. This suggests that novice students lack awareness of how the environment helps or hinders their tasks.

Table 1 summarizes several hypotheses that require further examination. Claims 1-8 are related to the empirical basis of design thinking theory, which guides the training of creative capabilities across the 4P domains. In design thinking, as in other areas, metacognition relies to a significant extent on beliefs about what is helpful and what is hindering in creative work. Therefore, it is crucial to ensure that the theoretical basis is correct. In contrast, hypotheses 9-15 relate to the impact of different educational measures on the development of creative metacognition.

**TABLE 1:** Hypotheses that invite more rigorous evaluation in future research.

1. The creativity of final work outcomes can be predicted even before creators start thinking about solution ideas, based on the task statements (project goals) they endorse. Task statements that consist of sentences no human has said or thought before, which involve unprecedented goals that seek meaningful solutions (e.g., to address unmet basic needs), predict greater creative achievements than task statements that are common, use often-employed success metrics or ask for random, less-meaningful solutions.
2. Experienced creators (design thinkers) are better at predicting the creativity of final work outcomes based on task statements compared to novice creators.
3. Experienced creators (design thinkers) have a more nuanced understanding of the different phases and their specific goals in the creative process compared to novices. A more nuanced understanding of the process predicts greater creative performance.
4. Given the same creative task, people achieve more highly creative work outcomes when their process involves an exploration of both problem and solution spaces, compared to people who only explore the solution space.
5. Collaboration in multi-perspective teams, along with the exploration of different perspectives across social groups, enhances long-term creative performance, even though it may require extra communication efforts in the short term.
6. Given a specific creative task, creators who can list more favorable versus hindering impacts of their work environment show enhanced task performance compared to creators who fail to consider environmental impacts on their work.
7. Creators who more often adapt their work environment to current objectives in their project, by rearranging the environment or moving to a different place, show enhanced creative performance compared to those who work in a static work environment.
8. If a person can identify a more diverse range of potential benefits and harms that a new design may cause across various areas of life, then they are more likely to produce highly creative products compared to a person who can only anticipate fewer or less diverse impacts.
9. Project-based creativity training that involves teamwork results in greater improvement in creative metacognition than individual work.
10. When working on creative projects for the same amount of time, interdisciplinary/multi-cultural teams show a more significant improvement in creative metacognition compared to mono-disciplinary/mono-cultural teams.
11. Making thinking explicit (such as by articulating thinking during multi-perspective teamwork) or tangible (such as by working with prototypes) improves metacognition among novices and experts.
12. A distinction between cognition and metacognition, where novices concentrate on creative cognition and experts take on metacognitive roles, leads to better development of metacognition among novices compared to a teaching approach that lacks this distinction.
13. Coaching interventions only when necessary (when markedly negative developments occur in any of the monitored domains) have a more favorable impact on the development of metacognition among trainees compared to regular coaching interventions.
14. Both the distribution of cognition and metacognition across time and space, as well as the distribution across people positively impact the development of metacognition in novice creators, compared to training approaches that use no such distinction.
15. Metacognition on People and Processes develops prior to metacognition on Products and Places (in current western education systems).

## 9. Next steps in the measurement of creative metacognition: from accuracy scores to more comprehensive assessments

As highlighted throughout the paper, creative metacognition is a complex phenomenon that serves numerous purposes in supporting creativity. When using the concept of creative metacognition as a variable in psychological research, it is essential to employ advanced measurement techniques to fully capture the phenomenon. In this regard, we will begin by summarizing a common assessment procedure to date and then, in Section “9.1. Beyond conformity to expert opinion: the importance of nuanced accuracy scores,” suggest refined metrics and interpretations based on a case example. Section 9.2 proposes a variety of further measurement approaches to assess creative metacognition in more comprehensive ways.

A common approach to measuring creative metacognition is to calculate accuracy scores that enable a comparison between an individual’s creative confidence and their actual performance (e.g., Kaufman et al., 2016; Karwowski et al., 2020; Puente-Díaz and Cavazos-Arroyo, 2022). This measure is straightforward in the case of well-defined problems, which have a single correct answer or a clearly defined performance dimension. For instance, examinees may be asked to estimate their own performance with regard to certain math problems. The personal estimate is then compared to the examinee’s true performance score when they actually work on such problems. A high level of accuracy – meaning that the estimate aligns with the actually achieved score – is a good sign for the individual’s metacognition. When a person can accurately judge their own performance, they are in the best position to take reasonable corrective actions if needed (cf. Kaufman et al., 2016 and Section “5. Creative metacognition on people”). For example, if an individual is weak in certain math problems and they are aware of it, they can practice more.

By contrast, creative problems do not have a single correct solution or a clearly defined performance dimension (Sections “1. Introduction” and “2. Design thinking as a practical approach to solving creative (ill-defined) problems”). In general, creative solutions are novel and valuable (Runco and Jaeger, 2012; Paul and Kaufman, 2014; Royalty and Roth, 2016). With regard to novelty, it may be possible to find objective performance metrics, such as statistical infrequency (Hocevar, 1979). However, with regard to value, this is not the case. Cultural groups may agree on certain evaluation criteria and their order of priority in assessing creative solutions at a given point in time. Yet, it is a hallmark of high-level breakthrough creativity that it changes these assessments, leading to new perspectives on what is important and desirable in a given field. This type of creativity results in paradigm shifts (Kuhn, 1962) and is referred to as Transformational Creativity, which alters people’s cognitive spaces (Boden, 2004). It leads to significant changes in the ways how people develop ideas and select solutions henceforth.

When it comes to creative problems, performance can be objectively quantified once a value dimension is established. For example, evaluators may require that a new solution in a particular field must be as inexpensive or lightweight as possible. However, it is impossible to claim that the chosen value dimensions for optimizing performance are the ultimate and best choices (Section “4. Creative metacognition on processes”). High-level breakthrough creativity often involves identifying value dimensions that were previously overlooked by the expert community, which however become increasingly important to communities later. For example, the managers of a car manufacturing company may agree that new motor design A is better than B because it has more horsepower. However, other stakeholders may hold different values, leading to paradigm shifts. From their perspective, sustainability may be the most important criterion and more relevant than horsepower (Hula et al., 2022). Exploring the experiences and viewpoints of diverse stakeholders can be an effective strategy to determine the worthiness of any given set of evaluation criteria (Section “4. Creative metacognition on processes”).

Against this background, interpreting accuracy scores in the context of creative metacognition requires caution. This contrasts to studies of metacognition that address well-defined problems, where the interpretation of accuracy is straightforward.

### 9.1. Beyond conformity to expert opinion: the importance of nuanced accuracy scores

A sample study that exemplifies current standards in the calculation of accuracy scores has been conducted by Kaufman et al. (2016). The authors let 242 elementary school children respond to creative challenges in the verbal, visual and scientific domain. Subsequently, four undergraduate students served as quasi-experts who evaluated the creativity of the children’s responses. In the data analysis, accuracy scores provided a basis for estimating metacognitive performance. “The goal of this study was to examine whether students had the metacognitive ability to judge their creative performance on a task *such that their ratings would correspond with expert ratings*” (2016, p. 395, emphasis added).

According to this approach, students could improve their metacognitive performance by learning to arrive at the same creativity judgments as the expert evaluators. However, in light of the metacognitive competencies listed in the 4P framework above, an approach like this may be counterproductive. It implicitly or explicitly introduces creativity experts as authorities who determine the creative merit of ideas, while it seems that students should accept the expert evaluations. Such an approach conflicts with the metacognitive learning goals in the 4P framework. For instance, in the process domain, design thinking trainees learn to overcome traditional, authority-directed work approaches (Section “4. Creative metacognition on processes”). In the people domain, trainees learn to empathize with diverse perspectives and to develop an internal locus of judgment (Section “5. Creative metacognition on people”). Similarly, Beghetto (2021) argues that emulating expert or teacher judgments may stifle the student’s creative exploration and potentially decrease their overall creative performance.

*[B]y the time students leave school, it is likely that they have learned that classroom success is less about providing their own creative ideas and more about figuring out the quickest and most direct route to produce what their teacher expects to hear (…).*

*School success therefore becomes more of a guessing game (i.e., guess what your teacher wants) than an opportunity to share and receive feedback on one’s own ideas and insights.*

*(Beghetto, 2021, p. 113)*

The potential dangers of adhering too closely to prevailing expert opinions can also be seen in the history of art. A notable example is Vincent van Gogh, now considered one of the most creative painters of all time. However, he lived in poverty and struggled to sell his works during his lifetime, as the public did not see value in his unique approach to painting (Kieran, 2014; Agnoli et al., 2019). If van Gogh had been told to improve his metacognitive appraisals by conforming to the evaluation standards of recognized experts at the time, it is likely that he would not have developed his unique style that is highly valued today.

Against this background, we suggest further differentiations in the use of accuracy scores when assessing creative achievements and metacognition. Specifically, we propose that two aspects of accuracy should be considered. When reviewers evaluate the product of a creator…

(I) How well can the creator predict the evaluation outcomes of different reviewers?

(II) To what extent does the creator agree with the reviewers, coming to the same evaluation outcome?

*Metric I* is easy to interpret and highly relevant. It measures performance in *theory of mind* (cf. Section “4. Creative metacognition on processes,” specifically Plank et al., 2021). Creators excel in this metric when they are able to understand the perspectives of different reviewers. As a methodological adjustment, we propose that reviewers from various stakeholder groups evaluate the creativity of test answers in research. For example, the same creative solution could be evaluated by economists, environmental activists, legal experts, artists, and advocates of gender equality, among others. Creators may be able to predict the creativity judgements of some expert groups better than those of others.

By contrast, interpreting *Metric II* is less straightforward and may present challenges. From a design thinking perspective, it is not desirable for students to generate novel ideas and select options in the exact same manner as their teachers or other experts (Section “4. Creative metacognition on processes”). With such an approach, the creator would stay “inside the box” of the expert community, missing opportunities of exploring grounds beyond. Ideally, in the case of breakthrough creativity, the student would come up with a solution that eventually sets new standards (Kuhn, 1962; Boden, 2004; Plattner et al., 2009), teaching the teacher new criteria that they did not initially consider. Therefore, a discrepancy between the student’s and the teacher’s evaluation is not inherently problematic. However, to indicate high levels of creative performance, the student’s evaluation criteria should be different *and* better (Arnold, 1959/2016). One potential method for measuring this is to allow for a space of argumentation. When the student can convince their teachers and potentially other experts or peers of the merits of their solution approach, the situation is to be treated differently compared to a student who may overestimate their performance in a narcissistic manner (the concern of Kaufman et al., 2016), or a student who suffers from blind spots in their evaluation.

### 9.2. Leveraging measurement opportunities in the service of comprehensive assessments

Although accuracy scores can be easily measured and refined by differentiating between metric I and II, such pinpointed measurement approaches do not fully capture the complexity of creative metacognition. Especially in applied settings such as schools or businesses, we need to be cautious about content validity (American Psychological Association, 1954), construct validity (Cronbach and Meehl, 1955) and ethical implications (Messick, 1980) when attempting to measure “creative metacognition,” rather than one of its many sub-constructs. Against such a background, we will highlight several methodological opportunities for measuring the construct of creative metacognition more comprehensively, particularly when considering the various facets of the phenomenon reviewed in Sections “3. Creative metacognition on products” up to “6. Creative metacognition on places.”

#### 9.2.1. Double-diamond-tasks with iteration

Selecting appropriate tasks is crucial when evaluating creative metacognition. To investigate the influence of metacognition on creative performance, it is important to place participants in situations that require its use. Ideally, tasks should be chosen where metacognitive reflections have the maximum impact on final task performance. We recommend experimental tasks in which participants are asked to explore a problem space before turning to solution spaces, following the double diamond model of creation (see Section “4. Creative metacognition on processes”). Moreover, it is highly desirable to include at least one round of iteration. According to Flavell (1987), a primary purpose of metacognition is to reassess what has been achieved, correct errors, and adjust strategies for future courses of action. When participants are asked to tackle a creative task and simply solve it, they may engage primarily in creative cognition. However, when they are instructed to tackle a creative task, reassess their approach and its outcomes, and then try again in a second round of iteration, it is this iterative step that requires metacognition. The difference between task outcomes in round 1 versus round 2 should directly reflect the impact of better versus worse metacognitive abilities. A bad design outcome in round 1 may even be a good thing if the creator is able to learn from mistakes, submitting a much improved solution in round 2 (cf. von Thienen et al., 2017, on the importance and opportunity of learning from failure in the creative process). Design thinking provides easy-to-understand instruction sheets that support novice creators in exploring problem spaces first, followed by an exploration of the selected solution space, and a round of iteration.^3^ These resources may offer inspiration for designing extended tasks to assess creative metacognition.

#### 9.2.2. 4P knowledge tests

The significance of knowledge has always been clear in the discussion of metacognition (Flavell, 1987; Papaleontiou-Louca, 2003; Martinez, 2006). Against this background, we propose that knowledge tests can play a more significant role in the assessment of creative metacognition. We specifically recommend evaluating expertise in each of the 4P domains: creative ***P***roducts, ***P***rocesses, ***P***eople, and ***P***laces. For example, in the domain of creative people, the examinee should demonstrate an understanding of the importance of originality/novelty, and the relationship between fluency in the process and final product creativity. In the domain of processes, the examinee may be asked to enumerate various methods they know for a specific purpose, such as generating novel ideas or evaluating them (cf. Arnold, 1959/2016, or d.school, 2010, for various methods that could be used).

#### 9.2.3. Combining naturalistic observations with controlled competency tests

A concern expressed in creativity research at large is the need for more naturalistic observations to increase ecological validity (Rafner et al., 2022; Jaschek et al., 2023). Some studies already move in this direction, such as Lefford and Thompson (2018). We want to emphasize the possibility of combining naturalistic observations with controlled competency tests and quantifications. In particular, such assessments can focus on the metacognition-flexibility relationship. This assessment approach traces the creator’s ability to reflect on their decisions made in a project, the reasons for each choice, and the awareness of alternative options. The examinee may be presented with a video recording of their own creative work, such as a 5-min clip. The video should depict their natural behavior while working on a real-life creative project. The examinee may then be tasked with identifying their decisions made during the recorded scene, and list as many potential alternatives as they can within a specified response time, such as 3 min. With adequate training, creators can become capable of recognizing numerous choices and alternatives, enabling them to make informed decisions and overcome obstacles in their creative process. For instance, an expert creator may state: “My team created the prototype of a novel vehicle in the form of a pencil sketch. Alternatively, we could have built a 3D model. We could have used a different process, for instance using machine intelligence or asking colleagues instead of following our own intuition. We could have designed the prototype with a different user audience in mind, such as elderly people rather than rich automobile lovers. We chose to go about this project in the spirit of product design; alternatively, we could have conducted an art project. We decided to create a rough prototype instead of developing a refined model…” When asked to provide reasons for each choice, well-trained creators can provide answers that are supported by theory and research. For instance, the examinee might state: “We chose to build a rough rather than a refined prototype, because we are just beginning to probe solution ideas, and we first want to test the gist of a concept before getting caught up in the details.” Such an explanation is in line with empirical evidence on the impact of using rough versus refined prototypes in creative thinking processes (Edelman and Currano, 2011). Furthermore, well-trained creators may be better aware of the alternatives they have for making particular choices. By way of an example, asked to enlist as many alternatives as possible for their pencil-sketch prototype, the expert examinee might state: “My team could have created a rough prototype by using Styrofoam, clay, any other objects like fruits,… or we could have done a role-play, or we could have used story-telling” (for different prototyping approaches, see McKim, 1972; Plattner et al., 2009; d.school, 2010; Edelman and Currano, 2011). Overall, such an assessment method combines the use of naturalistic creative behavior captured via video-recordings with time-constrained competency tests of reflecting on choices and their alternatives, as well as the reasons creators are able to provide for particular choices.

#### 9.2.4. Physiological stress measurement

Metacognition, as defined by Flavell (1987), encompasses the ability to organize support for error correction, helps in overcoming difficulties, and facilitates weighty decision-making in high-stake situations. In real-world scenarios, creators often encounter forms of hardship along the process, such as experiences of failure (Arnold, 1959/2016; Roth, 2015; von Thienen et al., 2017; Corazza and von Thienen, 2021). Emotion regulation in difficult situations, as when searching for novel and valuable solutions in a creative task and failing to find them, has been found to be a predictor of final creative achievement (Agnoli et al., 2019). Psycho-physiological models of how people cope with difficult situations emphasize the importance of (perceived) “resources” and “demands” in determining an individual’s stress response both on a cognitive and physiological level (Tomaka et al., 1993, 1997; Blascovich et al., 2003). Individuals with ample resources to address a demanding situation are better equipped to manage stressors and ultimately perform more creatively, while those with limited resources may feel overwhelmed and threatened by the task, resulting in a decrease in creative performance (Akinola et al., 2019). As previously discussed, creative metacognition involves being aware of alternative options. Therefore, metacognition can be a crucial resource that helps creators cope with difficult situations. In particular, demanding situations where initial decisions have led into a dead end may elicit greater stress responses among creators with low metacognitive performance (i.e., those who are unaware of potential alternative actions and do not believe in their creative growth abilities) compared to creators with high metacognitive performance (who can easily consider various alternative approaches to try next). Additionally, metacognition has been directly linked to emotion regulation – for example, when individuals are able to use metacognition as a means to re-appraise their stress experience (Papaleontiou-Louca, 2003; Martinez, 2006; Crum et al., 2017; Kim, 2019). Against this background, physiological stress indicators such as skin conductance, cardiovascular parameters, or cognitive load (Seery, 2013; Akinola et al., 2019) may serve as useful indirect metrics of metacognition, particularly when assessed in situations where creators face setbacks, for instance when their initial ideas meet with criticism from expert evaluators or other audiences. To establish this relationship of variables, studies should ideally collect physiological stress metrics alongside more direct measures of metacognitive performance. Moreover, conducting observations on multiple levels of analysis, such as gathering data on both the physiological and behavioral plane, helps to study the phenomenon of creative metacognition more comprehensively.

## 10. Conclusion

The need for increased focus on creative metacognition, both in terms of the number of studies conducted and the methodological challenges that must be addressed, is rightly emphasized by Puente-Díaz and Cavazos-Arroyo (2022). Their analysis, along with that of Sidi et al. (2020), is insightful in pointing out that multi-solution problems are inherent to creative work, which in turn poses difficulties when it comes to measuring creative metacognition.

In this paper, we introduce a comprehensive theoretical framework of creative metacognition. It aims to improve the basis for comprehensive assessments of creative metacognition competencies.

Overall, we discuss creative metacognition as thinking about creative thinking. It serves the purpose of improving creative performance, or enhancing creative capacity. Creative metacognition can manifest itself in four different areas, concerning creative ***P***roducts, ***P***rocesses, ***P***eople, and ***P***laces.

Creative metacognition on products refers to the monitoring and control of both problem and solution characteristics in a project. This includes project goals, task statements, evaluation criteria, and success metrics, as well as the novelty, value, and task appropriateness of ideas, prototypes, or final work outcomes. By monitoring the strengths and weaknesses of these elements and assisting with improvements, metacognition on products guides creators toward setting ambitious project goals and developing highly creative solutions characterized by high levels of novelty and value.

Creative metacognition on processes refers to the monitoring and control of activities as well as strategies used during the creative process, which can be optimized in order to arrive at the best possible creative outcomes. It also entails the ability to differentiate between various phases in the creative process, each characterized by different objectives, allowing the creator(s) to select and adjust their procedure according to the phase they are in.

Creative metacognition on people involves monitoring the strengths and weaknesses of individuals and groups engaged in a creative project, regulating their participation, and developing personal or collective strengths while addressing weaknesses. It helps with orchestrating and developing human resources in the creative project to arrive at the best possible creative outcomes.

Creative metacognition on places involves monitoring the strengths and weaknesses of environments for creative projects and for living creatures in general. It encompasses the control of work environments to enhance creative performance, as well as the control of creativity to maintain a livable planet.

In terms of practical facilitation, this paper presents three major approaches for enhancing creative metacognition in training contexts. The first approach involves distributing cognition and metacognition over time and space, highlighting the role of tools, time-boxing, and supportive room setups for the development of creative metacognition. The second strategy involves distributing cognition and metacognition across people, emphasizing the use of distinct roles, social support systems, and role models. The third strategy aims to make thinking manifest or tangible, providing clear content for reflection. This approach emphasizes the role of explicit thinking and tangible representations of thought as means to facilitate creative metacognition. While these approaches are commonly used in design thinking education and some of them have been discussed favorably in the creative metacognition literature (Section “7. Strategies of educating for creative metacognition in design thinking”), more empirical research is needed to quantify their impact.

Furthermore, the 4P framework of creative metacognition includes competencies such as recognizing weaknesses in authority-directed work approaches (Section “4. Creative metacognition on processes”) or understanding and predicting idea assessments of different stakeholder groups (Section “5. Creative metacognition on people”). Against this background, we have critically discussed the use of accuracy scores in creative metacognition research, emphasizing questions of measurement validity and the goal of guarding against potential negative effects on examinees. These issues can arise when selected experts act as authorities who determine the creative merit of ideas. To address these concerns, we propose using more nuanced accuracy scores. Metric I measures performance in theory of mind by having examinees predict idea evaluations of different stakeholder groups. Metric II compares the individual’s assessment of their creative idea to assessments made by “experts,” providing opportunities for argumentation.

This paper invites researchers to think more critically about content, construct and ethical validity when assessing creative metacognition. From the perspective of design thinking, metacognitive competencies need to be measured across all 4P domains. We have started to identify key metacognitive competencies across these fields. In terms of methodologies for measuring creative metacognition more comprehensively, we suggest *Double-Diamond-Tasks with Iteration* in which examinees explore problem and solution spaces, including at least one round of iteration, *4P Knowledge Tests* in which examinees are tested on their knowledge of methods, cause-effect relationships, and presumed strengths or weaknesses in the 4P domains, *Combinations of Naturalistic Observations with Controlled Competency Tests* in which creators reflect on their choices and alternative options when reviewing their own creative behavior, as well as *Physiological Stress Measurement* in which the examinee’s skin conductance or other physiological parameters are obtained during situations of failure or hardship in creative work.

One important aspect that requires more attention in studies of creative metacognition is the role of knowledge or beliefs, specifically regarding perceived strengths and weaknesses in creative endeavors. In this context, it is important to consider the validity of claims regarding cause-effect relationships. For instance, is a creative process that focuses solely on exploring solution spaces a weakness in creative work? Does such a process predict lower levels of creative performance compared to an approach that involves the exploration of both problem and solution spaces? Design thinking students are taught that this is the case – in line with hypothesis 4 in Table 1. After training, metacognitive reflections and presumed error corrections may prompt design thinking students to explore problem spaces in addition to solution spaces. However, for metacognitive competencies to be effective, the underlying theoretical basis must be predominantly accurate. Therefore, it is essential for creativity research and research on creative metacognition to collaborate closely. We need to develop valid and comprehensive frameworks that can effectively measure and facilitate creative (meta-)cognition in education and practice.

## Data availability statement

The original contributions presented in this study are included in the article/supplementary material, further inquiries can be directed to the corresponding author.

## Author contributions

JT wrote a draft of the manuscript and undertook revisions. TW wrote sections of the manuscript especially related to practices at the HPI D-School and physiological measurement approaches, and revised the manuscript. CM was involved in conceptualizing the framework. All authors contributed to the article and approved the submitted version.

## Footnotes

1
https://dschool.stanford.edu/resources/gear-up-how-to-kick-off-a-crash-course

2
https://github.com/mollyclare/design-dash

3 https://dschool.stanford.edu/resources/gear-up-how-to-kick-off-a-crash-course; https://dschool.stanford.edu/s/Participant-Worksheet.pdf; https://dschool.stanford.edu/s/1TheGiftGivingProjectFacilitatorsGuide-English.pdf; https://github.com/mollyclare/design-dash; Stanford Design Thinking Virtual Crash Course: https://www.youtube.com/watch?v=pmjyZPibH14
